# microRNAs involved in the control of toxicity on locomotion behavior induced by simulated microgravity stress in *Caenorhabditis elegans*

**DOI:** 10.1038/s41598-020-74582-z

**Published:** 2020-10-15

**Authors:** Lingmei Sun, Wenjie Li, Dan Li, Dayong Wang

**Affiliations:** Medical School, Southeast University, Nanjing, 210009 China

**Keywords:** Environmental impact, Biomarkers

## Abstract

microRNAs (miRNAs) post-transcriptionally regulate the expression of targeted genes. We here systematically identify miRNAs in response to simulated microgravity based on both expressions and functional analysis in *Caenorhabditis elegans*. After simulated microgravity treatment, we observed that 19 miRNAs (16 down-regulated and 3 up-regulated) were dysregulated. Among these dysregulated miRNAs, *let-7*, *mir-54*, *mir-67*, *mir-85*, *mir-252*, *mir-354*, *mir-789*, *mir-2208*, and *mir-5592* were required for the toxicity induction of simulated microgravity in suppressing locomotion behavior. In nematodes, alteration in expressions of *let-7*, *mir-67*, *mir-85*, *mir-252*, *mir-354*, *mir-789*, *mir-2208*, and *mir-5592* mediated a protective response to simulated microgravity, whereas alteration in *mir-54* expression mediated the toxicity induction of simulated microgravity. Moreover, among these candidate miRNAs, *let-7* regulated the toxicity of simulated microgravity by targeting and suppressing SKN-1/Nrf protein. In the intestine, a signaling cascade of SKN-1/Nrf-GST-4/GST-5/GST-7 required for the control of oxidative stress was identified to act downstream of *let-7* to regulate the toxicity of simulated microgravity. Our data demonstrated the crucial function of miRNAs in regulating the toxicity of simulated microgravity stress in organisms. Moreover, our results further provided an important molecular basis for epigenetic control of toxicity of simulated microgravity.

## Introduction

During the spaceflight, microgravity will cause several aspects of adverse effects on human beings. In humans, the alterations in central aortic blood pressure, motor performance, muscle function, and metabolism could be detected under the microgravity condition^1–5^. Model animal of *Caenorhabditis elegans* shows a high sensitivity to stresses or toxicants^6,7^. It was employed as an assay animal in ‘‘the first International *C. elegans* Experiment in Space’’ (ICE-First) experiments to determine possible toxic effects on animals during the spaceflight^8–10^. Based on spaceflight experiments, microgravity treatment at least potentially affected reproduction, locomotion behavior, early embryogenesis, and gene expression in nematodes^8,9,11–14^.

Simulated microgravity treatment is an important strategy to examine the microgravity effects on animals or humans. In humans, simulated microgravity treatment could result in psychic performance, headache, and abnormal endocrine^15,16^. Recently, the toxicity of simulated microgravity could also be detected in nematodes^17–19^. Simulated microgravity could induce production of intestinal reactive oxygen species (ROS) and inhibition in locomotion behavior in nematodes^20^. Meanwhile, insulin, p38 mitogen-activated protein kinase (MAPK), and Wnt signaling pathways were required for toxicity induction in simulated microgravity treated nematodes^19–21^. Nevertheless, the molecular responses of organisms to simulated microgravity still remain largely unknown.

*Caenorhabditis elegans* is an important animal model for the study of both molecular toxicology and target organs toxicity of environmental toxicants or stresses^22,23^. Meanwhile, *C. elegans* is suitable for determining the long-term effects from treatment with certain toxicants or stresses^7,23^. It has been supposed that 1-day in nematodes is comparable to 4.2-year in humans^24^. The short noncoding RNAs of microRNAs (miRNAs) can regulate post-transcriptionally the expression and the functions of many targeted genes^25,26^, which represents a kind of epigenetic regulation mechanism for the control of gene expression^27,28^. In nematodes, some miRNAs have been identified to be involved in the control of response to environmental toxicants, such as graphene oxide (GO)^22,23,29,30^. The molecular mechanisms for certain miRNAs in regulating the response to toxicants have also been elucidated^22,23,31,32^. Some of previous studies have tried to systematically identify the miRNAs in response to microgravity or simulated microgravity only based on the sequencing data^33–36^. We here used *C. elegans* to systematically identify miRNAs involved in the control of toxicity of simulated microgravity stress on locomotion behavior based on both expressions and functional analysis. Moreover, we focused on the *let-7* to determine the underlying mechanism for its role in regulating the toxicity of simulated microgravity. Our data provided the molecular basis for our understanding the miRNAs-mediated epigenetic control of toxicity of simulated microgravity in nematodes.

## Results

### Identification of dysregulated miRNAs by simulated microgravity treatment

**Figure 1:**
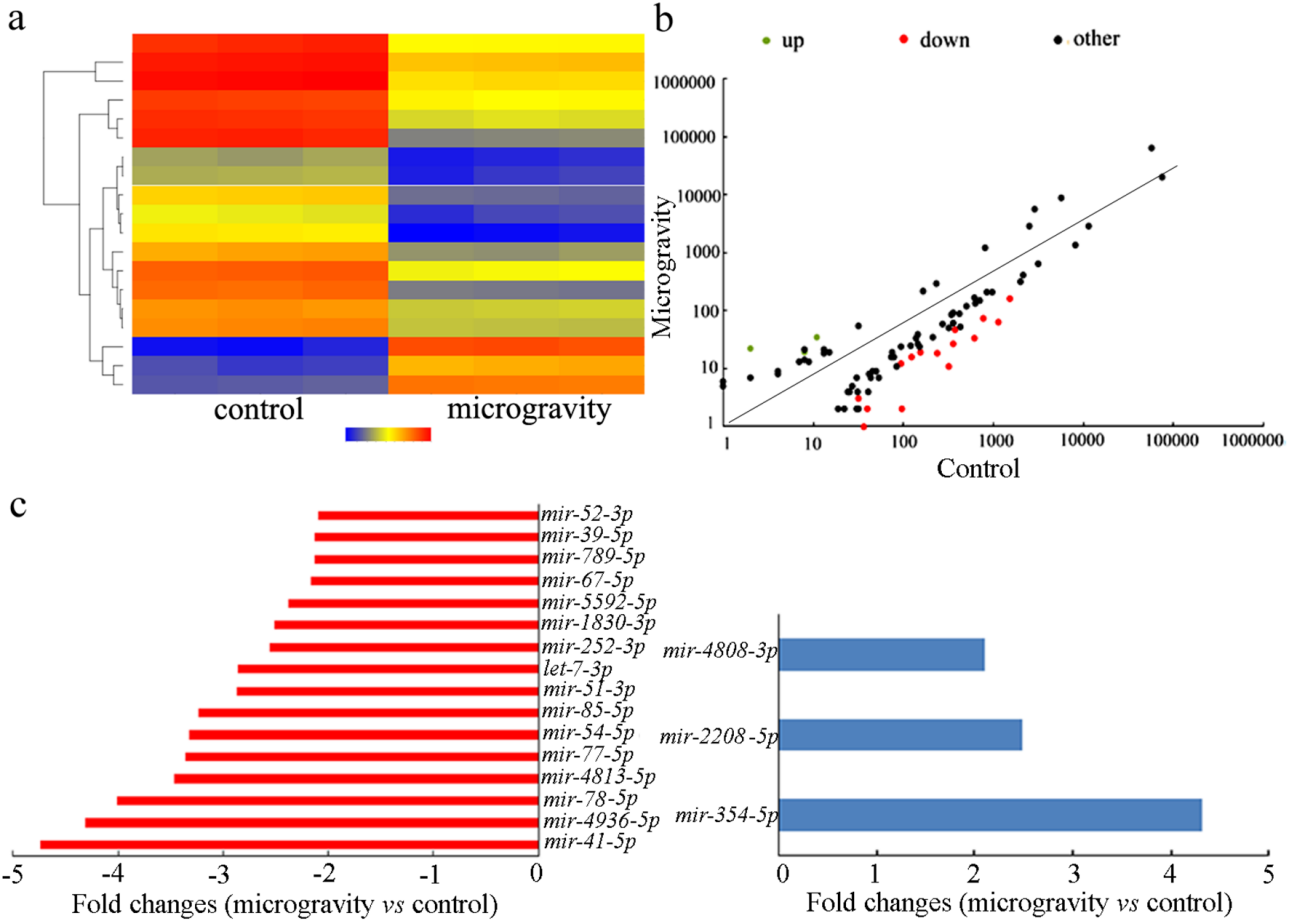
Dysregulation of microRNAs by simulated microgravity treatment in wild-type nematodes. (**a**) Heat map of identified dysregulated microRNAs in wild-type nematodes after simulated microgravity treatment. (**b**) Scatter diagram of miRNAs coverage of the control group and the simulated microgravity treatment group. (**c**) Down-regulated and up-regulated microRNAs in wild-type nematodes after simulated microgravity treatment. Simulated microgravity treatment was performed in RCCS system at 30 rpm and for 24 h.

After simulated microgravity treatment in RCCS system at 30 rpm and for 24 h, we identified 19 dysregulated miRNAs based on the SOLiD sequencing (Fig. 1 and Table S1). Among these 19 dysregulated miRNAs, 3 up-regulated miRNAs and 16 downregulated miRNAs were identified (Fig. 1 and Table S1). The up-regulated miRNAs contained *mir-4808*, *mir-2208*, and *mir-354*, and the downregulated miRNAs contained *mir-52*, *mir-39*, *mir-789*, *mir-67*, *mir-5592*, *mir-1830, mir-252*, *let-7*, *mir-85*, *mir-77*, *mir-4813*, *mir-78*, *mir-4936*, *mir-54*, *mir-51*, and *mir-41* in simulated microgravity treated animals (Fig. 1 and Table S1).

### Effect of *mir-67, mir-77, mir-78, mir-85, mir-252, mir-52, mir-51,* or* let-7* mutation on toxicity of simulated microgravity

**Figure 2:**
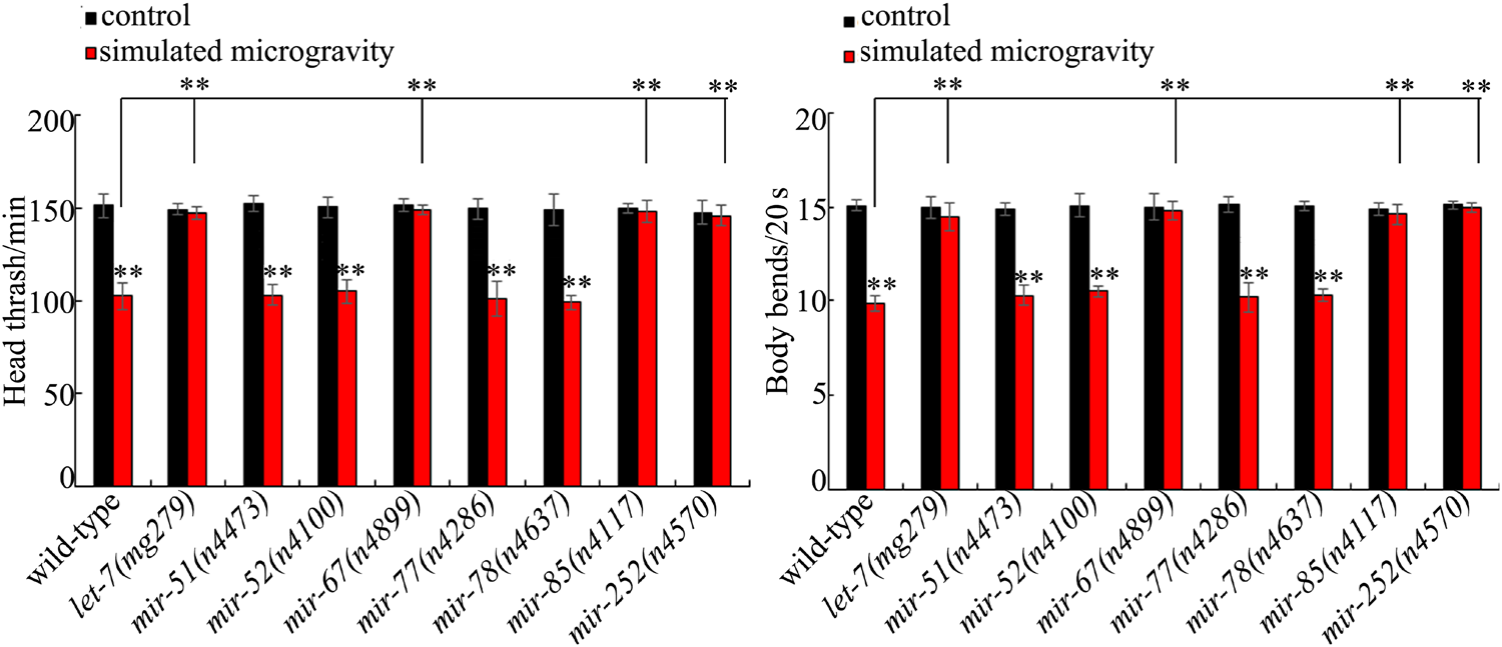
Effect of *mir-51*, *mir-52*, *mir-67*, *mir-77*, *mir-78*, *mir-85*, *mir-252*, or *let-7* mutation on toxicity of simulated microgravity in decreasing locomotion behavior in nematodes. Simulated microgravity treatment was performed in RCCS system at 30 rpm and for 24 h. Bars represent means ± SD. **P < 0.01 *vs* control (if not specially indicated).

Among the 19 candidate miRNAs, the genetic mutants for 8 miRNAs (*mir-51*, *mir-52*, *mir-67*, *mir-77*, *mir-78*, *mir-85*, *mir-252*, and *let-7*) are available. To confirm the role of these miRNAs in affecting the toxicity of simulated microgravity, we investigated the effects of *mir-67*, *mir-77*, *mir-78*, *mir-85*, *mir-252*, *mir-52*, *mir-51*, or *let-7* mutation on locomotion behavior in simulated microgravity treated nematodes. Under the normal conditions, the *mir-67*, *mir-77*, *mir-78*, *mir-85*, *mir-252*, *mir-52*, *mir-51*, or *let-7* mutants did not affect the locomotion behavior (Fig. 2). After the treatment, mutation of *mir-51*, *mir-52*, *mir-77*, or *mir-78* did not influence toxicity of simulated microgravity in inhibiting locomotion behavior (Fig. 2). In contrast, we observed the noticeable suppression in toxicity on locomotion behavior in simulated microgravity treated *let-7*, *mir-67*, *mir-85*, or *mir-252* mutants compared with simulated microgravity treated wild-type animals (Fig. 2).

### Effect of* mir-789, mir-5592, mir-1830, mir-54, mir-4813, mir-4936, mir-41, mir-4808, mir-2208, mir-39, or mir-354* overexpression on toxicity of simulated microgravity

**Figure 3:**
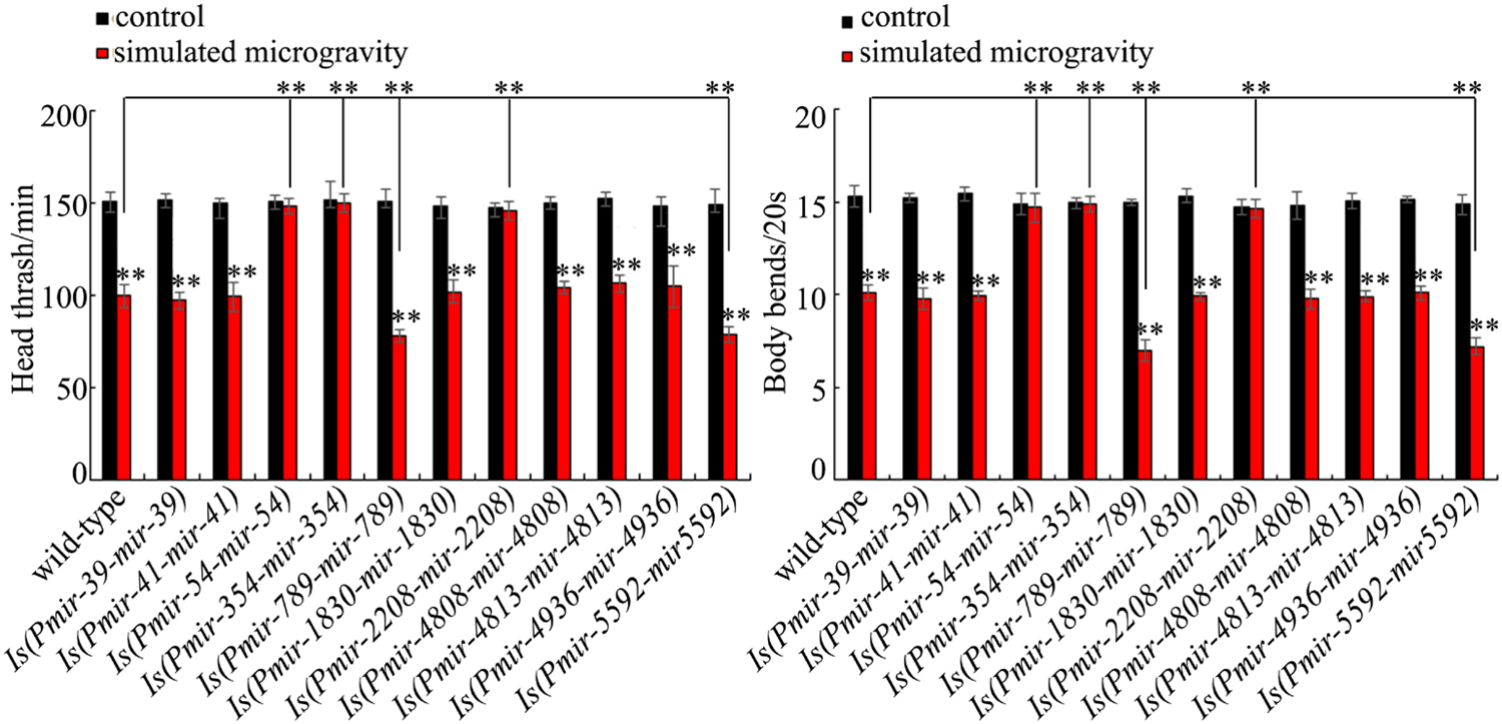
Effect of *mir-39*, *mir-789*, *mir-5592*, *mir-1830*, *mir-54*, *mir-4813*, *mir-4936*, *mir-41*, *mir-4808*, *mir-2208*, or *mir-354* overexpression on toxicity of simulated microgravity in decreasing locomotion behavior in nematodes. Simulated microgravity treatment was performed in RCCS system at 30 rpm and for 24 h. Bars represent means ± SD. **P < 0.01 *vs* control (if not specially indicated).

For the other 11 candidate miRNAs, we generated transgenic nematode strains overexpressing these miRNAs. Under the normal conditions, nematodes overexpressing *mir-39*, *mir-789*, *mir-5592*, *mir-1830*, *mir-54*, *mir-4813*, *mir-4936*, *mir-41*, *mir-4808*, *mir-2208*, or *mir-354* did not show the obvious alteration in locomotion behavior (Fig. 3). We observed that overexpression of *mir-39, mir-1830*, *mir-4813*, *mir-4936*, *mir-41*, or *mir-4808* did not obviously affect the toxicity of simulated microgravity on locomotion behavior (Fig. 3). In contrast, we detected more severe suppression in locomotion behavior in simulated microgravity treated nematodes overexpressing *mir-789* or *mir-5592* compared with simulated microgravity treated wild-type animals (Fig. 3). In addition, overexpression of *mir-54*, *mir-354*, or *mir-2208* suppressed the toxicity on locomotion behavior in simulated microgravity treated animals (Fig. 3). Therefore, our data further suggested the functions of *mir-54*, *mir-354*, *mir-789*, *mir-2208*, and *mir-5592* in regulating the toxicity of simulated microgravity.

### qRT-PCR conformation of the effect of simulated microgravity on expressions of candidate miRNAs

**Figure 4:**
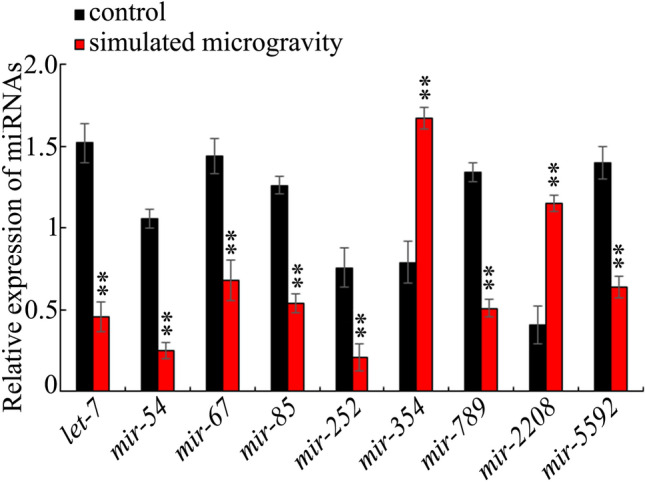
qRT-PCR analysis of microRNAs expression in simulated microgravity treated wild-type nematodes. Simulated microgravity treatment was performed in RCCS system at 30 rpm and for 24 h. Bars represent means ± SD. **P < 0.01 *vs* control.

Using qRT-PCR technique, we observed that the simulated microgravity in RCCS system at 30 rpm and for 24 h significantly decreased expressions of *mir-54*, *mir-67*, *mir-85*, *mir-789*, *mir-252*, *let-7*, and *mir-5592* (Fig. 4). Additionally, the simulated microgravity could further significantly increase the expressions of *mir-354* and *mir-2208* (Fig. 4).

### Biological processes mediated by 9 candidate miRNAs based on gene ontology analysis

Based on the gene ontology analysis, the possible biological processes mediated by *mir-54*, *mir-67*, *mir-85*, *mir-252*, *mir-354*, *mir-789*, *mir-2208*, *let-7*, and *mir-5592* were classified into the categories of development, reproduction, cellular localization, cellular organization, cellular adhesion, cell proliferation, metabolism, and rhythmic process (Fig. S1 and Table S2). Additionally, these 9 candidate miRNAs were also associated with the control of immune response and response to stimulus (Fig. S1 and Table S2).

### *let-7* acted in both intestine and neurons to regulate the toxicity of simulated microgravity

**Figure 5:**
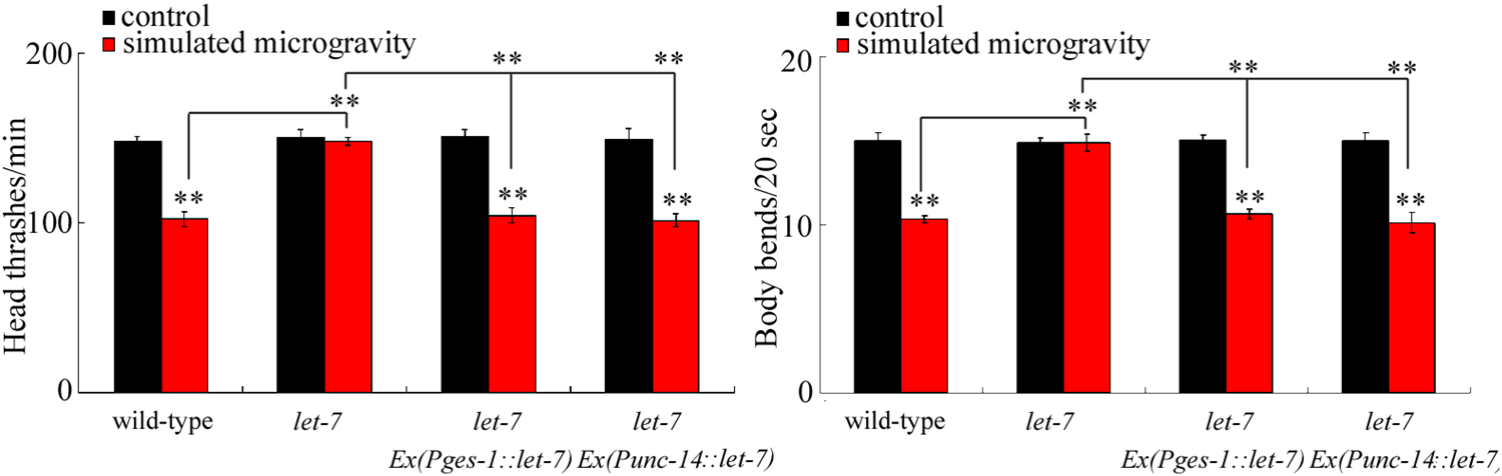
*let-7* acted in both intestine and neurons to regulate the toxicity of simulated microgravity in decreasing locomotion behavior in nematodes. Simulated microgravity treatment was performed in RCCS system at 30 rpm and for 24 h. Bars represent means ± SD. **P < 0.01 *vs* control (if not specially indicated).

We further focused on *let-7* to determine the underlying mechanism for its role in regulating the toxicity of simulated microgravity. Previous study has indicated that the *let-7* could function in both the intestine and the neurons to regulate the response to environmental toxicants or stresses^24,37^. We further observed that intestinal overexpression of *let-7* (*Ex(*P*ges-1::let-7)*) caused the significant decrease in locomotion behavior in simulated microgravity treated *let-7* mutant nematodes (Fig. 5). Similarly, neuronal overexpression of *let-7* (*Ex(*P*unc-14::let-7)*) also resulted in the significant decrease in locomotion behavior in simulated microgravity treated *let-7* mutant nematodes (Fig. 5). Therefore, the *let-7* could act in both the intestine and the neurons to regulate the toxicity of simulated microgravity.

### Genetic interaction between *let-7* and SKN-1a or SKN-1b in regulating the toxicity of simulated microgravity

**Figure 6:**
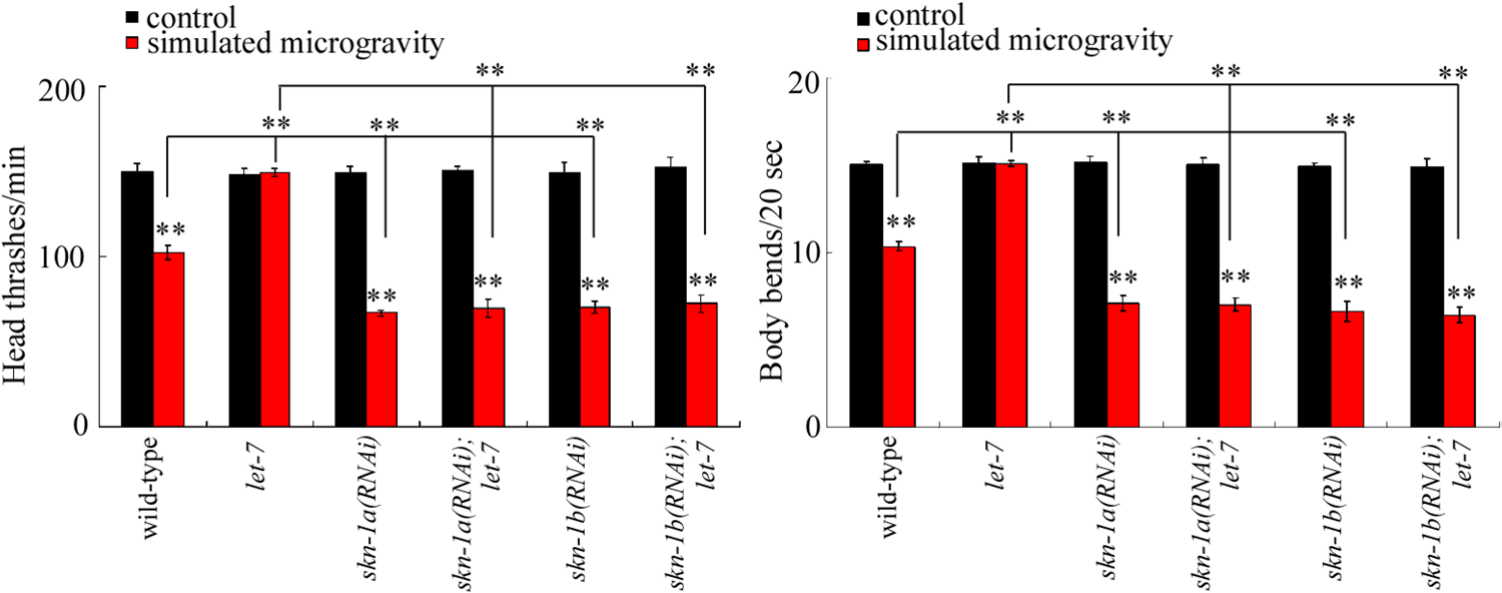
Genetic interaction between *let-7* and SKN-1a or SKN-1b in regulating the toxicity of simulated microgravity in decreasing locomotion behavior in nematodes. Simulated microgravity treatment was performed in RCCS system at 30 rpm and for 24 h. Bars represent means ± SD. **P < 0.01 *vs* control (if not specially indicated).

Our previous study has demonstrated that the oxidative stress acted as a crucial contributor to the toxicity in decreasing locomotion behavior in simulated microgravity treated nematodes^17^. Among the predicted targets using TargetScan (https://www.targetscan.org/worm_52/), SKN-1/Nrf is a bZip transcriptional factor required for the control of oxidative stress response^38^. During the control of innate immunity, previous study has indicated the role of SKN-1 as the direct target of *let-7* family of miRNAs^39^. In nematodes, the SKN-1a is expressed in the intestine, and the SKN-1/b is expressed in the neurons. RNA interference (RNAi) knockdown of *skn-1a* or *skn-1b* enhanced the toxicity of simulated microgravity in decreasing locomotion behavior, suggesting the susceptibility of *skn-1a(RNAi)* or *skn-1b(RNAi)* nematodes to neurotoxicity of simulated microgravity (Fig. 6). Moreover, RNAi knockdown of *skn-1a* or *skn-1b* significantly inhibited the resistance of *let-7* mutant nematodes to toxicity of simulated microgravity in decreasing locomotion behavior (Fig. 6), suggesting that both the SKN-1a and the SKN-1b acted as downstream targets of *let-7* in regulating the toxicity of simulated microgravity.

### Genetic interaction between intestinal SKN-1a and some GST proteins in regulating the toxicity of simulated microgravity

**Figure 7:**
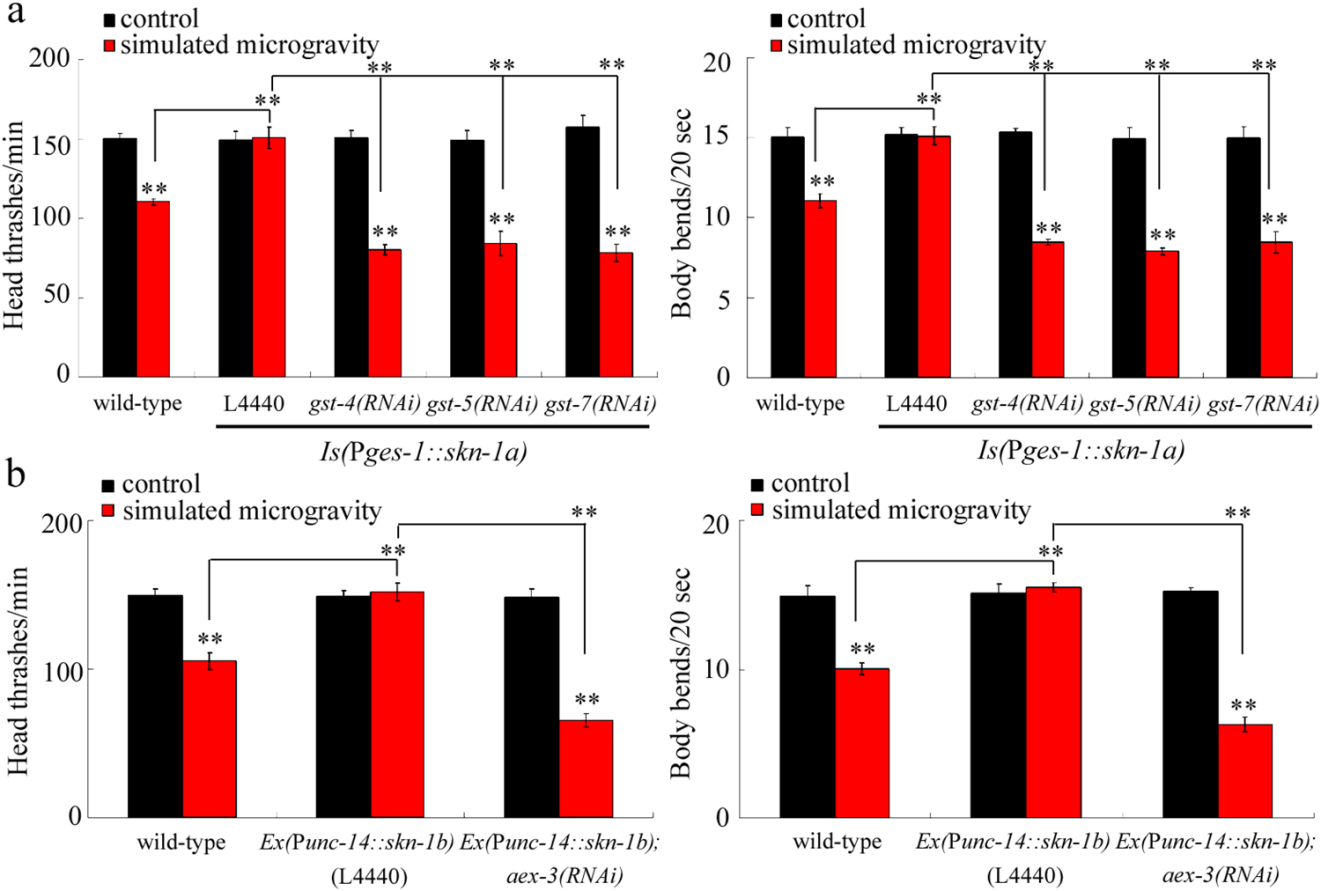
Identification of downstream targets of SKN-1a and SKN-1b in regulating the toxicity of simulated microgravity in decreasing locomotion behavior in nematodes. (**a**) Genetic interaction between SKN-1a and GST-4, GST-5, or GST-7 in regulating the toxicity of simulated microgravity. (**b**) Genetic interaction between SKN-1b and AEX-3 in regulating the toxicity of simulated microgravity. L4440, empty vector. Simulated microgravity treatment was performed in RCCS system at 30 rpm and for 24 h. Bars represent means ± SD. **P < 0.01 *vs* control (if not specially indicated).

SKN-1 normally regulates the oxidative stress by targeting glutathione-S-transferases^38,40^. Some glutathione-S-transferases (GST-4, GST-5, and GST-7) have been identified as the targets of intestinal SKN-1 in regulating the stress response^24,40^. Nematodes (*Is(*P*ges-1::skn-1a)*) overexpressing intestinal SKN-1a showed the suppressed toxicity of simulated microgravity in decreasing locomotion behavior (Fig. 7a), suggesting the resistance of *Is(*P*ges-1::skn-1a)* nematodes to toxicity of simulated microgravity. Moreover, we found that RNAi knockdown of *gst-4*, *gst-5*, and *gst-7* all could significantly inhibit the resistance of *Is(*P*ges-1::skn-1a)* nematodes to toxicity of simulated microgravity in decreasing locomotion behavior (Fig. 7a). Thus, GST-4, GST-5, and GST-7 acted as downstream targets of intestinal SKN-1a to regulate the toxicity of simulated microgravity.

Nematodes (*Is(*P*unc-14::skn-1b)*) overexpressing neuronal SKN-1b also exhibited the suppressed toxicity of simulated microgravity in decreasing locomotion behavior (Fig. 7b), suggesting the resistance of *Is(*P*unc-14::skn-1b)* nematodes to toxicity of simulated microgravity. Previous study has suggested the role of AEX-3, a guanine exchanger factor for GTPase, as the target of SKN-1b in regulating the response to toxicants, such as GO^41^. Moreover, RNAi knockdown of *aex-3* could further significantly suppress the resistance of *Is(*P*unc-14::skn-1b)* nematodes to toxicity of simulated microgravity in decreasing locomotion behavior (Fig. 7b). Therefore, the AEX-3 acted as a downstream target of neuronal SKN-1b to regulate the toxicity of simulated microgravity.

### Genetic interaction between* let-7* and its targets in regulating the toxicity of simulated microgravity in inducing ROS production

**Figure 8:**
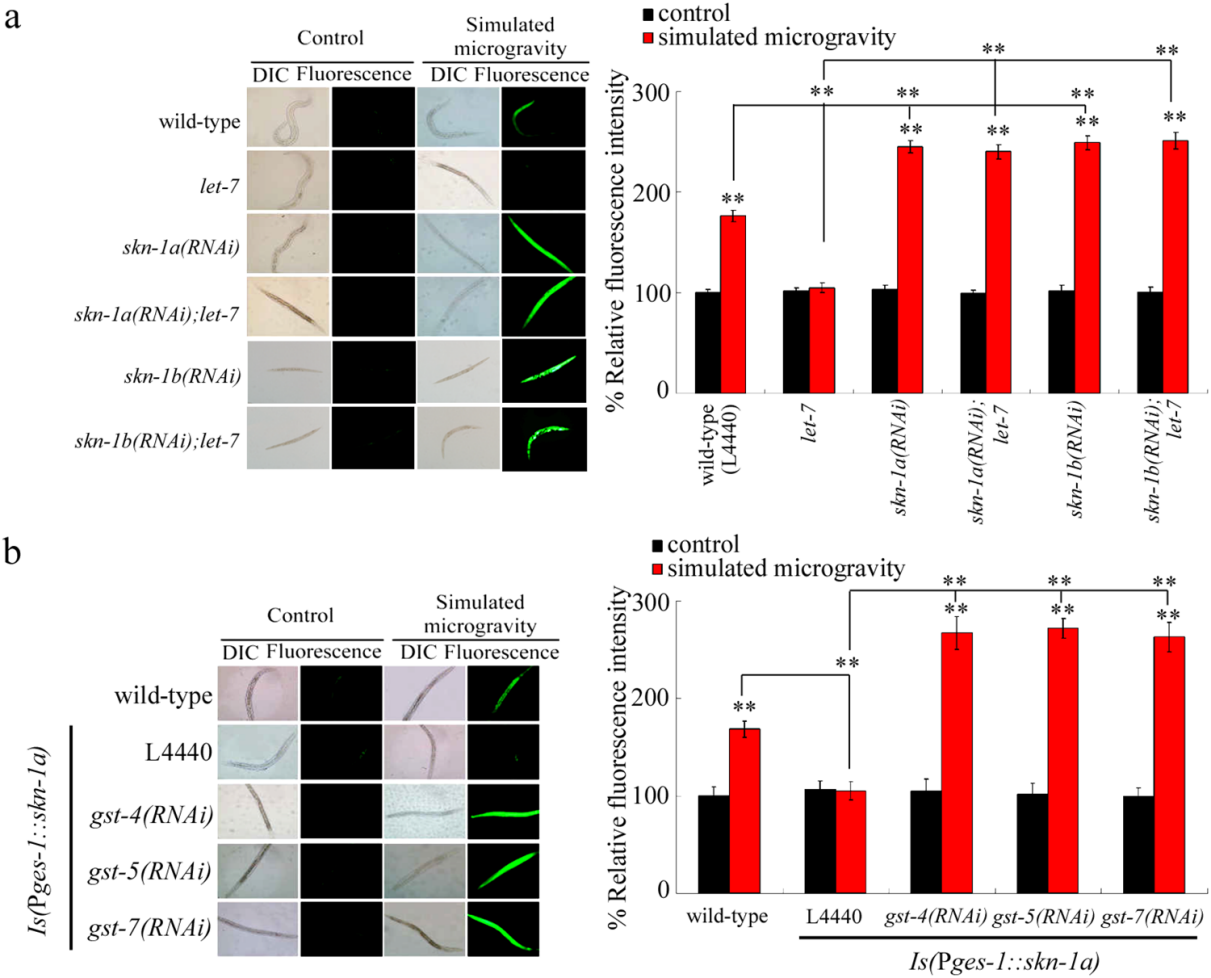
*let-7* and its targets regulated the toxicity of simulated microgravity in inducing ROS production in nematodes. (**a**) Genetic interaction between *let-7* and SKN-1a or SKN-1b in regulating the toxicity of simulated microgravity in inducing ROS production. (**b**) Genetic interaction between intestinal SKN-1a and GST-4, GST-5, or GST-7 in regulating the toxicity of simulated microgravity in inducing ROS production. L4440, empty vector. Simulated microgravity treatment was performed in RCCS system at 30 rpm and for 24 h. Bars represent means ± SD. **P < 0.01 *vs* control (if not specially indicated).

Mutation of *let-7* could suppress the induction of ROS production in simulated microgravity treatment nematodes (Fig. 8a), suggesting the resistance of *let-7* mutant to toxicity of simulated microgravity in activating oxidative stress. Genetic interaction analysis further indicated that RNAi knockdown of *skn-1a* inhibited the resistance of *let-7* mutant to toxicity of simulated microgravity in inducing ROS production (Fig. 8a). Additionally, RNAi knockdown of *skn-1n* also suppressed the resistance of *let-7* mutant to toxicity of simulated microgravity in inducing ROS production (Fig. 8a).

Moreover, RNAi knockdown of *gst-4*, *gst-5*, or *gst-7* suppressed the resistance of *Is(*P*ges-1::skn-1a)* nematodes overexpressing intestinal SKN-1a to the toxicity of simulated microgravity in inducing ROS production (Fig. 8b). That is, in the intestine, GST-4, GST-5, and GST-7 could act as the downstream targets of intestinal SKN-1a to regulate the activation of oxidative stress in simulated microgravity treated nematodes.

## Discussion

*Caenorhabditis elegans* is a powerful tool to determine expression and functions of miRNAs, and some works have implied involvement of miRNAs during regulating response to stresses or toxicants^7,36,37^. In this study, we identified 19 dysregulated miRNAs caused by simulated microgravity treatment in RCCS system at 30 rpm and for 24 h (Fig. 1 and Table S1). Previous study has examined the dysregulated miRNAs during the Shenzhou-8 mission spaceflight^35^. Nevertheless, the identified dysregulated miRNAs by simulated microgravity in this study were largely different from those by Shenzhou-8 mission spaceflight. This may be largely due to the dysregulation in miRNAs caused by both environmental irradiation and microgravity during the Shenzhou-8 mission spaceflight.

Using the available mutants, we observed the resistance of *let-7*, *mir-67*, *mir-85*, or *mir-252* mutant nematodes to the toxicity of simulated microgravity (Fig. 2). Using transgenic strains, we further found the susceptibility of nematodes overexpressing *mir-789* or *mir-5592* and the resistance of nematodes overexpression of *mir-54*, *mir-354*, or *mir-2208* to the toxicity of simulated microgravity (Fig. 3). Therefore, we provided direct and functional evidence to indicate the important functions of some miRNAs (*mir-54*, *mir-67*, *mir-85*, *mir-252*, *mir-354*, *mir-789*, *mir-2208*, *let-7*, and *mir-5592*) in regulating the toxicity of simulated microgravity. Among these miRNAs, the *let-7* was involved in the control of response to toxicants (such as multi-walled carbon nanotubes) or innate immune response to bacterial infection^37,42^. The *mir-252* was required for the control of innate immune response to fungal infection^43^.

Among the confirmed 9 miRNAs involved in regulating the neurotoxicity of simulated microgravity, meanwhile, simulated microgravity could decrease the expressions of *mir-54*, *mir-67*, *mir-85*, *mir-252*, *mir-789*, *let-7*, and *mir-5592*, and increase the expressions of *mir-354* and *mir-2208* (Fig. 1 and Table S1). The expressions of these 9 candidate miRNAs in simulated microgravity could be confirmed by qRT-PCR analysis (Fig. 4). These results suggested that alteration in expressions of *mir-67*, *mir-85*, *mir-252*, *mir-354*, *mir-789*, *mir-2208*, *let-7*, and *mir-5592* mediated a protective response to simulated microgravity. In contrast, the alteration in *mir-54* expression mediated the neurotoxicity induction of simulated microgravity. Therefore, the dsyregulated miRNAs may mediate two different responses to simulated microgravity in nematodes. Previous study has also suggested that, in GO exposed nematodes, alterations in expression of some miRNAs (such as *mir-247*) mediated the toxicity induction, whereas alterations in expression of some other miRNAs (such as *mir-231*) mediated a protective response to GO toxicity^29,44,45^, which further supports the observed dual responses of miRNAs to certain stresses or toxicants in organisms.

In this study, gene ontology analysis demonstrated that *mir-54*, *mir-67*, *mir-85*, *mir-252*, *mir-354*, *mir-789*, *mir-2208*, *let-7*, and *mir-5592* mediated a subset of biological processes (Fig. S1 and Table S2). The data of bioinformatical analysis provides important clues for understanding the possible roles and functions of these 9 miRNAs in response to microgravity stress.

microRNAs act in conserved post-transcriptional gene regulatory mechanism in various organisms^46^. Based on the sequence relationships, the homologues of human miRNAs for *C. elegans let-7* include *let-7a-let-7i*, *miR-98*, *miR-196*, and *miR-196b*, the homologues of human miRNAs for *C. elegans mir-54* include *miR-99a*, *miR-99b*, and *miR-100*, and the homologues of human miRNAs for *C. elegans mir-252* include *miR-26a* and *miR-26b*^46^. Thus, the detected dysregulated miRNAs in nematodes raised useful clues to understand functions of some miRNAs in response to microgravity stress in humans. Previous study has indicated that the modeled microgravity could dysregulate the expressions of *let-7i* and *let-7e* in human peripheral blood hymphocytes^36^. Spaceflight could affect the expressions of *let-7* family (*let-7i*, *let-7f.*, *let-7c,* and *let-7a*) in human fibroblast cells^33^. The simulated microgravity could also affect *let-7a* expression in human skeletal muscles^34^. These results further support our assumption on functions of certain number of miRNAs in regulating the response to microgravity stress based on the observations in nematodes.

Based on both the response of *let-7* to simulated microgravity in nematodes and the response of *let-7* family to microgravity in human cell lines introduced above, we focused on the *let-7* to determine the underlying mechanism for its role in regulating the toxicity of simulated microgravity. In nematodes, the *let-7* could function in both intestine and the neurons to regulate the toxicity of simulated microgravity (Fig. 5). Meanwhile, the genetic interaction analysis implied that the SKN-1a acted as the downstream target of *let-7* in the intestine to regulate the toxicity of simulated microgravity, and the SKN-1b acted as the downstream target of *let-7* in the neurons to regulate the toxicity of simulated microgravity (Fig. 6). Therefore, the neuronal signaling cascade of *let-7*-SKN-1b may directly regulate the toxic effect of simulated microgravity on locomotion behavior. Among the identified miRNAs, *mir-54* and *mir-252* are also expressed in the neurons (https://wormbase.org//). The *mir-54* and *mir-252* and their targets may also possibly act in the neurons to regulate the toxic effect of simulated microgravity on locomotion behavior.

Moreover, RNAi knockdown of *aex-3* could suppress the resistance of nematodes overexpressing neuronal SKN-1b to toxicity of simulated microgravity (Fig. 7b). Therefore, during the control of toxicity of simulated microgravity, we raised a neuronal signaling cascade of SKN-1b-AEX-3. Besides this, we further found that RNAi knockdown of *gst-4*, *gst-5*, or *gst-7* could inhibit the resistance of nematodes overexpressing intestinal SKN-1a to toxicity of simulated microgravity (Fig. 7a). That is, during the control of toxicity of simulated microgravity, we also raised an intestinal signaling cascade of SKN-1a-GST-4/GST-5/GST-7. Our previous study has suggested that the activation of PMK-1/p38 MAPK-SKN-1 mediated a protective response to simulated microgravity^20^. In this study, we further found that the decrease in *let-7* could further enhance the p38 MAPK signaling pathway-mediated protective response to simulated microgravity by targeting SKN-1 s. Our results in this study provided an important molecular basis for our understanding the epigenetic control of response to simulated microgravity in organisms.

Our previous study has demonstrated that treatment with antioxidant of ascorbate could prevent the neurotoxicity of simulated microgravity in decreasing locomotion behavior^17^, indicating the crucial role of oxidative stress as a contributor of neurotoxicity induction of simulated microgravity. Moreover, we found that *let-7* could regulate the toxicity of simulated microgravity in activating oxidative stress by targeting SKN-1a or SKN-1b (Fig. 8). Additionally, intestinal SKN-1a regulated the toxicity of simulated microgravity in activating oxidative stress by affecting the functions of some glutathione-S-transferases (GST-4, GST-5, and GST-7) (Fig. 8). Therefore, in the intestine, the signaling cascade of *let-7*-SKN-1a-GST-4/GST-5/GST-7 was raised to explain the molecular basis for the activation of oxidative stress induced by simulated microgravity.

Moreover, we found that the expressions of *mir-67*, *mir-252*, and *mir-5592* could also be decreased by simulated hypermicrogravity treatment (Fig. S2), suggested that both simulated microgravity and simulated hypergravity might activate the similar *mir-67*, *mir-252*, and *mir-5592*-mediaed responses. Nevertheless, the simulated hypermicrogravity treatment did not obviously affect the expressions of *let-7*, *mir-54*, *mir-85*, *mir-354*, *mir-789*, and *mir-2208* (Fig. S2).

Together, we employed *C. elegans* to examine the miRNAs involved in the control of toxicity of simulated microgravity on locomotion behavior. Using SOLiD sequencing technique, we identified 19 miRNAs in response to simulated microgravity treatment. Phenotypic analysis based on mutants and transgenic strains further suggested that 9 miRNAs (*mir-54*, *mir-67*, *mir-85*, *mir-252*, *mir-354*, *mir-789*, *mir-2208*, *let-7*, and *mir-5592*) were required for the control of toxicity of simulated microgravity on locomotion behavior. These 9 miRNAs mediated two different molecular responses for nematodes to simulated microgravity. Moreover, SKN-1a-GST-4/GST-5/GST-7 and SKN-1b-AEX-3 were identified as downstream signaling cascades of *let-7* in different tissues to regulate the toxicity of simulated microgravity. Our data highlights the crucial role of miRNAs in regulating the toxicity of simulated microgravity in nematodes.

## Methods

### *Caenorhabditis elegans* strains and maintenance

Besides the wild-type nematodes (N2), *mir-67(n4899)*, *mir-85(n4117)*, *mir-252(n4570), mir-78(n4637)*, *mir-77(n4286)*, *mir-52(n4100)*, *mir-51(n4473)*, and *let-7(mg279)* mutants, and transgenic strains of *Is(*P*ges-1::skn-1a)*^47^, *Is(*P*mir-39-mir-39)*, *Is(*P*mir-789-mir-789)*, *Is(*P*mir-5592-mir-5592)*, *Is(*P*mir-1830-mir-1830)*, *Is(*P*mir-54-mir-54)*, *Is(*P*mir-4813-mir-4813)*, *Is(*P*mir-4936-mir-4936)*, *Is(*P*mir-41-mir-41)*, *Is(*P*mir-4808-mir-4808)*, *Is(*P*mir-2208-mir-2208)*, *Is(*P*mir-354-mir-354)*, *let-7(mg279)Ex(*P*ges-1::let-7)*^37^, *let-7(mg279)Ex(*P*unc-14-::let-7)*^37^, and *Ex(*P*unc-14::skn-1b)*^41^ were used. Animals were grown on normal nematode growth mediate (NGM) plates, and fed with *Escherichia coli* OP50 (a food source) as described^48^.

### Simulated microgravity treatment

We performed the simulated microgravity treatment basically as described^49^. A soft and movable 0.2% agar medium was prepared for suspending animals in the assay chamber. In the cultivation chamber of the Rotary System (Synthecon) (Fig. S3), the vessels with the suspended young adults were half filled. We generated the simulated microgravity after balancing sedimentation-induced gravity with centrifugation by Rotary Cell Culture System (RCCS) vessel rotation^50^. RCCS rotated the chamber horizontally at 30 rpm for 24 h to set up simulated microgravity treatment. Young adults were used to perform the simulated microgravity treatment. Control nematodes were grown in soft and movable 0.2% agar medium without simulated microgravity treatment.

### Simulated hypergravity treatment

Nematode hypergravity cultivation tub was prepared according to previous protocols^51^. The 1 ml of NGM was placed into each 1.5 ml centrifuge tube and then transferred to a heat block set to prevent immediate solidification of agar. After distribution into tubes, the tubes were spun at 100G for 2 min to solidify the agar and create a surface in which the force of gravity is perpendicular to the flat agar surface in the tube. To create a small lawn of *E. coli* bacteria, a single colony of OP50 strain bacteria was inoculated in LB broth and incubated in a shaker overnight, then concentrated by spinning down and removing the supernatant. The pellet was re-suspended and 2 µl of bacteria was added to the surface of each tube and allowed to dry at room temperature for at least 24 h before usage. For hypergravity experiments, approximately 200 worms were placed into the cultivation tube, and centrifuged in a temperature-controlled micro-centrifuge (Centrifuge 5424R, Eppendorf) at 100G at 20 °C for 24-h. A control tube (1G) maintained in a 20 °C incubator was performed.

### Small RNA extraction and SOLiD sequencing

Control and simulated microgravity treatment groups were used for the exaction of small RNAs for the RNAomics analysis. mirVana miRNA isolation kit (Ambion) was used to isolate small RNAs, which were further used for preparing library of double-stranded cDNAs. After quality evaluation of library using Agilent 2100 Bioanalyzer, the library was used for high-throughput sequencing by Applied Biosystems SOLiD system. After comparison of nucleotide sequences obtained by SOLiD sequencing with miRNAbase and Genbank databases, registered miRNAs would be determined. The dysregulated miRNAs were judged by fold change analysis (2.0-fold change cutoff) together with statistical significance (*p* < 0.05).

### Quantitative real-time polymerase chain reaction (qRT-PCR)

Total RNAs were isolated using Trizol (Sigma-Aldrich). Synthesis of cDNAs with reverse transcriptase reaction was prepared by Mastercycler gradient PCR system (Eppendorf). Primer information for reverse transcription of miRNAs is shown in Table S3. Expression levels of examined miRNAs were analyzed by StepOnePlus real-time PCR system (Applied Biosystems) using SYBR Green qRT-PCR master mix (TOYOBO, Japan). *F35C11.9* encoding a small nuclear RNA U6 was used as a reference. Three replicates were carried out for the reactions. Table S4 shows the information of primers used for qRT-PCR.

### Locomotion behavior

Functional state of motor neurons was reflected by locomotion behaviors (body bend and head thrash)^52^. After treatment, nematodes were washed using M9 buffer first. A body bend refers to an alteration in direction of bending at the middle body. A head thrash refers to an alteration in direction of posterior bulb part. Forty nematodes were analyzed per treatment. Three replicates were performed.

### Induction of ROS production

Activation of oxidative stress was reflected by ROS production^53^. After the treatment, the animals were labeled with 1 µM CM-H_2_DCFDA for 3-h without light. After that, animals were examined at 510 nm of emission filter and at 488 nm of excitation wavelength using a laser scanning confocal microscope. Semi-quantification was analyzed for fluorescence intensity in comparison to autofluorescence using Image J software. Fifty animals were analyzed per treatment. Three replicates were performed.

### DNA constructs and germline transformation

The promoter of *mir-789*, *mir-5592*, *mir-1830*, *mir-54*, *mir-4813*, *mir-4936*, *mir-41*, *mir-4808*, *mir-2208*, *mir-39*, or *mir-354* was amplified from genome of wild-type animals. The promoter fragment was subcloned into pPD95_77 vector. After that, *mir-39*, *mir-789*, *mir-5592*, *mir-1830*, *mir-54*, *mir-4813*, *mir-4936*, *mir-41*, *mir-4808*, *mir-2208*, or *mir-354* was inserted into pPD_95_77 with its own promoter. Germline transformation was preformed by co-injecting the prepared DNA constructs (DNA (10–40 μg/mL) together with P*dop-1::rfp* (60 μg/mL) as marker DNA into the gonad^54^. Table S5 shows the information of primer used for preparation of DNA constructions.

### Bioinformatical analysis

For the candidate *mir-54*, *mir-67*, *mir-85*, *mir-252*, *mir-354*, *mir-789*, *mir-2208*, *let-7*, and *mir-5592*, gene ontology based on targets of these miRNAs were carried out by online bioinformatics analysis tool (https://www.pantherdb.org/). Potential targets of these miRNAs were predicted by online TargetScan database.

### RNAi assay

L1-larvae were fed with *E. coli* HT115 carrying double-stranded RNA corresponding to *skn-1a*, *skn-1b*, *aex-3*, *gst-4*, *gst-5*, or *gst-7* on NGM plates^55^. Before the growth on NGM plates, HT115 was transferred into LA broth (LB broth containing 100 μg/L ampicillin) with the addition of 5 mM isopropyl 1-thio-β-D-galactopyranoside (IPTG). After the development into gravid nematodes on RNAi plates, the nematodes were transferred to new RNAi plate to lay eggs. The second generation was used for exposure. HT115 expressing the empty vector L4440 was used as a control. Efficiency for RNAi knockdown of *skn-1a*, *skn-1b*, *aex-3*, *gst-4*, *gst-5*, or *gst-7* was determined by qRT-PCR (data not shown).

### Statistical analysis

SPSS 12.0 was used for statistical analysis. One-way analysis of variance (ANOVA) was used to determine the differences between groups. Two-way ANOVA analysis was used to determine multiple factor comparison. Probability level of 0.01 was considered statistically significant.

## Supplementary information

Supplementary information 1.
