# Supplementary material for: microRNAs involved in the control of toxicity on locomotion behavior induced by simulated microgravity stress in Caenorhabditis elegans
Source: Sci Rep. 2020 Oct 15;10:17510. doi: 10.1038/s41598-020-74582-z (PMC7567087; doi:10.1038/s41598-020-74582-z)
Supplement: Supplementary file 1 — Supplementary information 1. [file 41598_2020_74582_MOESM1_ESM.doc]

**microRNAs involved in the control of toxicity on locomotion behavior induced by simulated microgravity stress in *Caenorhabditis elegans***

Lingmei Sun, Wenjie Li, Dan Li, Dayong Wang^*^

Medical School, Southeast University, Nanjing 210009, China

^*^Correspondence and requests for materials should be addressed to D.W (dayongw@seu.edu.cn).

**Supporting Information:**


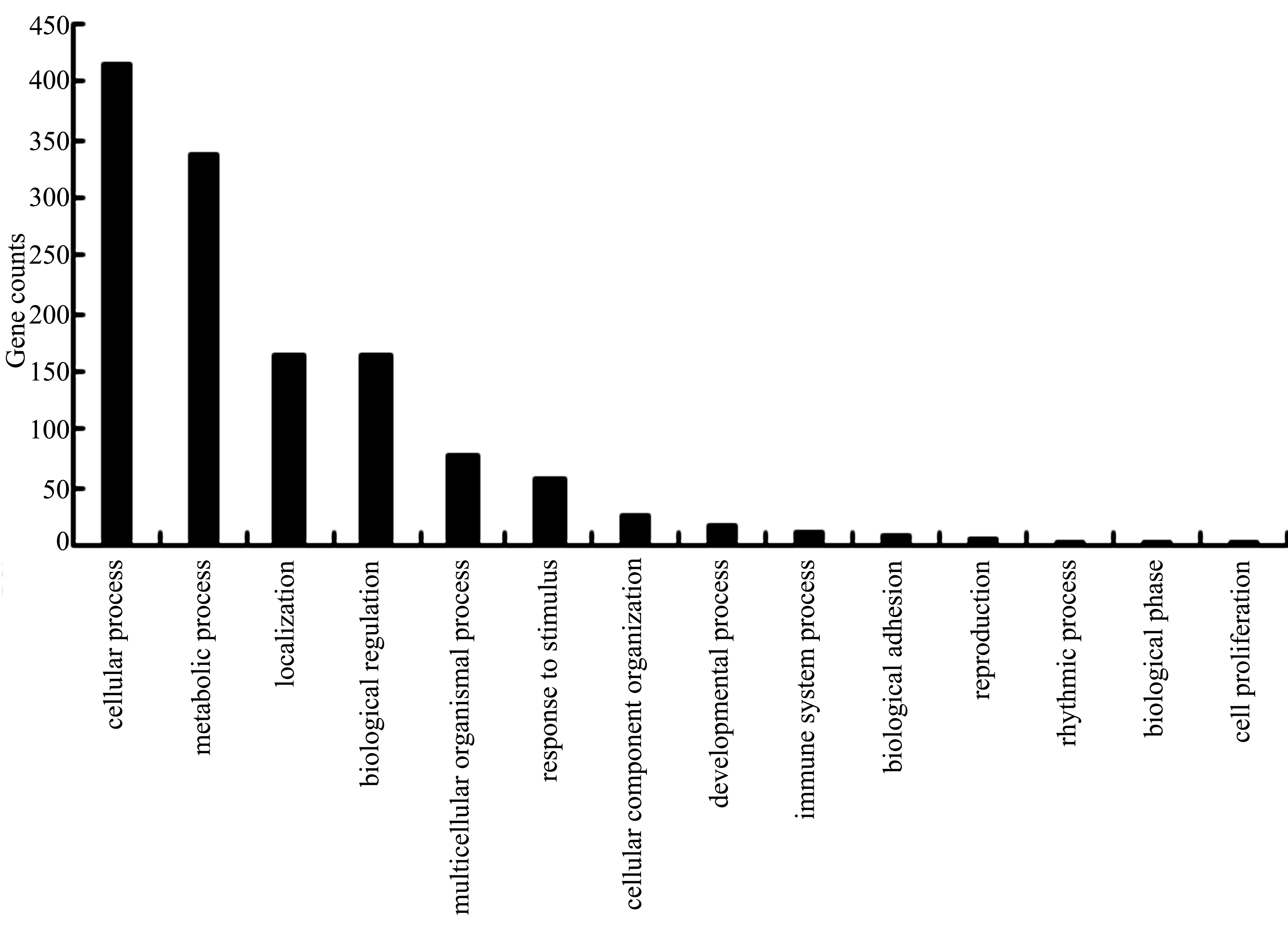


**Figure S1.** Analysis of gene ontology based on dysregulated miRNAs (*let-7*, *mir-54*, *mir-67*, *mir-85*, *mir-252*, *mir-354*, *mir-789*, *mir-2208*, and *mir-5592*) in simulated microgravity treated nematodes.

**
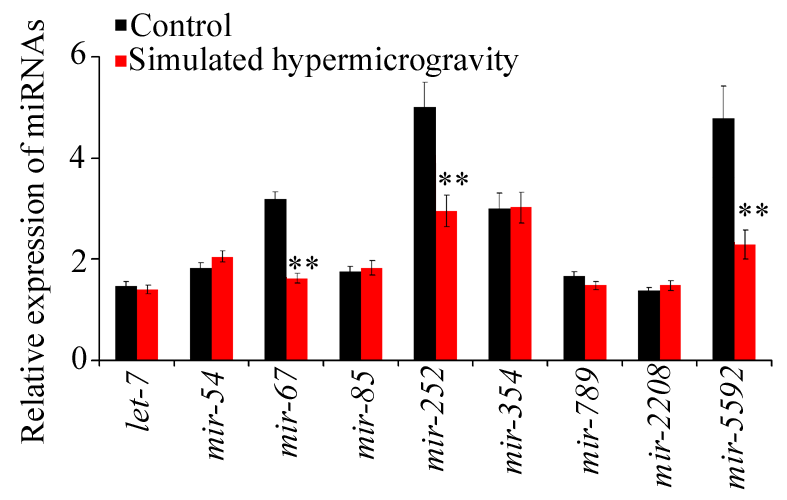
**

**Figure S2.** qRT-PCR analysis of microRNAs expression in simulated hypergravity treated wild-type nematodes. Simulated hypergravity treatment was performed at 100G and for 24 h. Bars represent means ± SD. ^**^*P* < 0.01 *vs* control.


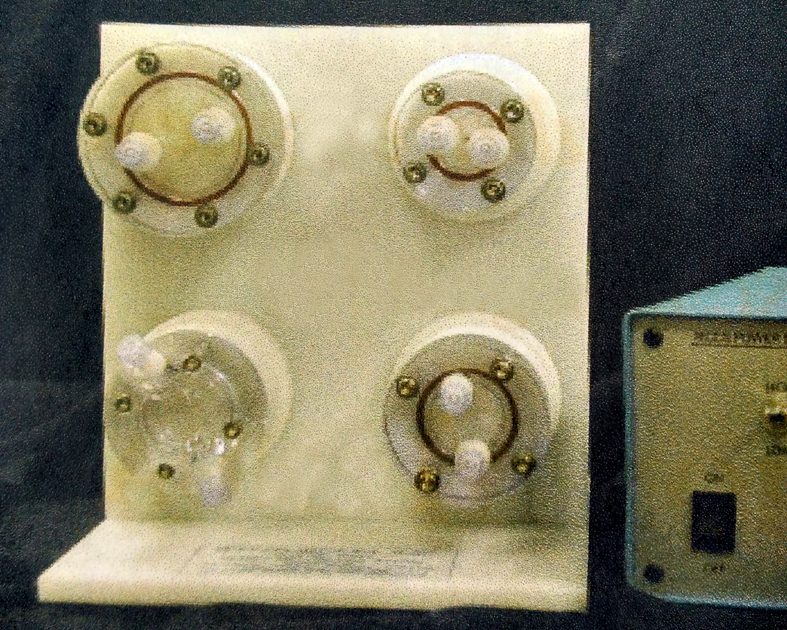


**Figure S3.** Rotary System™, which was developed by National Aeronautics and Space Administration (Washington, DC, USA) and manufactured by Synthecon (Houston, TX, USA).

**Table S1.** Dysregulated miRNAs by simulated microgravity

| ID | Accession | Fold changes  (FC) | Regulation |
| --- | --- | --- | --- |
| *MIMAT0020770* | *mir-41-5p* | -4.79657 | Down |
| *MI0017722* | *mir-4936-5p* | -4.36726 | Down |
| *MI0000049* | *mir-78-5p* | -4.03082 | Down |
| *MIMAT0020000* | *mir-4813-5p* | -3.47058 | Down |
| *MIMAT0020774* | *mir-77-5p* | -3.3779 | Down |
| *MIMAT0020773* | *mir-54-5p* | -3.22516 | Down |
| *MIMAT0020324* | *mir-85-5p* | -3.18746 | Down |
| *MIMAT0015099* | *mir-51-3p* | -2.87321 | Down |
| *MIMAT0015091* | *let-7-3p* | -2.86727 | Down |
| *MIMAT0020325* | *mir-252-3p* | -2.58931 | Down |
| *MIMAT0020778* | *mir-1830-3p* | -2.51244 | Down |
| *MIMAT0022305* | *mir-5592-5p* | -2.42537 | Down |
| *MIMAT0020316* | *mir-67-5p* | -2.38436 | Down |
| *MIMAT0032017* | *mir-789-5p* | -2.36935 | Down |
| *MIMAT0020306* | *mir-39-5p* | -2.31235 | Down |
| *MIMAT0020311* | *mir-52-3p* | -2.24234 | Down |
| *MIMAT0019992* | *mir-4808-3p* | 2.105502 | Up |
| *MIMAT0011429* | *mir-2208b-5p* | 2.485611 | Up |
| *MIMAT0031894* | *mir-354-5p* | 4.317001 | Up |

**Table S2.** Gene ontology terms based on dysregulated miRNAs (*let-7*, *mir-54*, *mir-67*, *mir-85*, *mir-252*, *mir-354*, *mir-789*, *mir-2208*, and *mir-5592*)

| No. | GO accession | GO term | Counts |
| --- | --- | --- | --- |
| 1 | GO:0009987 | cellular process | 417 |
| 2 | GO:0008152 | metabolic process | 337 |
| 3 | GO:0051179 | localization | 165 |
| 4 | GO:0065007 | biological regulation | 165 |
| 5 | GO:0032501 | multicellular organismal process | 77 |
| 6 | GO:0050896 | response to stimulus | 58 |
| 7 | GO:0071840 | cellular component organization | 26 |
| 8 | GO:0032502 | developmental process | 18 |
| 9 | GO:0002376 | immune system process | 11 |
| 10 | GO:0022610 | biological adhesion | 10 |
| 11 | GO:0000003 | reproduction | 7 |
| 12 | GO:0048511 | rhythmic process | 3 |
| 13 | GO:0044848 | biological phase | 2 |
| 14 | GO:0008283 | cell proliferation | 2 |

**Table S3.** Primers for reverse transcription of miRNAs

| miRNA | Primer |
| --- | --- |
| *let-7-3p* | GTCGTATCCAGTGCAGGGTCCGAGGTATTCGCACTGGATACGACGGTAAGG |
| *mir-54-5p* | GTCGTATCCAGTGCAGGGTCCGAGGTATTCGCACTGGATACGACTGTTCTC |
| *mir-67-5p* | GTCGTATCCAGTGCAGGGTCCGAGGTATTCGCACTGGATACGACCATAACA |
| *mir-85-5p* | GTCGTATCCAGTGCAGGGTCCGAGGTATTCGCACTGGATACGACGTTTCTT |
| *mir-252-3p* | GTCGTATCCAGTGCAGGGTCCGAGGTATTCGCACTGGATACGACGCAGAAG |
| *mir-354-5p* | GTCGTATCCAGTGCAGGGTCCGAGGTATTCGCACTGGATACGACATACCCG |
| *mir-789-5p* | GTCGTATCCAGTGCAGGGTCCGAGGTATTCGCACTGGATACGACGTCCTTG |
| *mir-2208b-5p* | GTCGTATCCAGTGCAGGGTCCGAGGTATTCGCACTGGATACGACGGATATC |
| *mir-5592-5p* | GTCGTATCCAGTGCAGGGTCCGAGGTATTCGCACTGGATACGACCATGTAT |

**Table S4**. Primers for real-time PCR of miRNAs

|  | primer |
| --- | --- |
| *let-7-3p* | CGGGCCCTATGCAATTTTCTAC |
| *mir-54-5p* | TGGTAGAGGATATGAGACGACG |
| *mir-67-5p* | ATTACGCGCTCATTCTGCCGGT |
| *mir-85-5p* | TCGTAGCCGATTTTTCAATAGT |
| *mir-252-3p* | ATTACGCTTACCTACTGCCTTC |
| *mir-354-5p* | ATTACGGGTGCGGCTGCAGACG |
| *mir-789-5p* | TGGTAGAATTGATGACCCAGAC |
| *mir-2208b-5p* | TGGTAGAAGTGTACCCGGATCT |
| *mir-5592-5p* | ATTACGCGGCCCTTACCGTTTA |
| Common reward primer | GTGCAGGGTCCGAGGT |
| *F35C11.9*/forward primer | GAAGATTAGCATGAACCC |
| *F35C11.9*/reverse primer | TTGGAACGCTTTATGAAT |

**Table S5. Primer information for DNA constructions**

|  | Forward primer (5’-3’) | Reverse primer (5’-3’) |
| --- | --- | --- |
| *Pmir-39* | CCGAAGCTTTTGATAAAAAGTTACGTC | CCACTGCAGTCAGGAATTCAATAATCA |
| *Pmir-41* | ATAAAGCTTCGGACTCGACACGACAGT | GGCCTGCAGACTTAAAAATAACTCACC |
| *Pmir-54* | ATACTGCAGTTGATACCAAAACAATTG | CCATCTAGAATTACCAATTCCAGAGAT |
| *Pmir-354* | ATATCTAGAGCGGAAATTAAACCAGCG | ATAGGTACCTTCCAAATTTCAGCCAAA |
| *Pmir-789* | GCCCTGCAGTTAAAAATTAAAATTTTT | ATAGGATCCTTCGTCTGATTCATACCG |
| *Pmir-1830* | ATAAAGCTTGGTGGCGGCGGCGTGCGA | GGCCTGCAGGCCACTCACCGACAGTGA |
| *Pmir-2208* | ACCAAGCTTTATCACGCCCACCTCTGT | ATACTGCAGACCACTGGCGGGTTCCTC |
| *Pmir-4808* | CGGCTGCAGTTGCTCAAACTAGTGGAC | ATACCCGGGCATGTTTAGCAATGCAAG |
| *Pmir-4813* | CCAAAGCTTGATATGGTTATCATCCGT | GCCCTGCAGAAAGAATAGAATAGTTGT |
| *Pmir-4936* | CCACTGCAGTAATTGTACTTTCTGCAA | ACCCCCGGGATATTAATAATTGAGAAT |
| *Pmir-5592* | CCGAAGCTTATGATTGACGAAGCTGAT | ATACTGCAGGCTGGGGCTTTACAACAT |
| *mir-39* | CATGGATCCTATACCGAGAGCCCAGCT | ATACCCGGGTTTCAGCTGAACATGGAA |
| *mir-41* | CGAGGATCCGGGTCCCAGAGACCTTGG | ATACCCGGGCCAAGGTCTCTGGGACCC |
| *mir-54* | ATACCCGGGGTGAGTCGCGCTCTGACT | CCACCCGGGCTGAAAGTTTTTCTGGAA |
| *mir-354* | ATAGGTACCCAGAGCCGACTAAGCACC | TATCTCGAGCAAAACCAATAGGAGCAG |
| *mir-789* | ATAGGATCCTGTCCTGAAGGCGGACAA | CGGACCGGTAAAAAAATTATTGCGTTG |
| *mir-1830* | ATACCCGGGCGAGGTTTCACGTTTTCT | GCCGGTACCGACAATTGTAAAATTCAG |
| *mir-2208* | ACCCTGCAGAAGTGTACCCGGATCTGA | ATAGGATCCTTTGAATTCCTCCGGCGT |
| *mir-4808* | ATACCCGGGGTAAGATAGATTAGTGCT | AACGGTACCAACAATTGATCAAATGAT |
| *mir-4813* | CCACTGCAGATTTCACCAGACTATCTA | ATAGGATCCTCGGAAAAACTGGAATAT |
| *mir-4936* | CCACCCGGGTATACGCTATCGAGATAA | ATAGGTACCAGGCCACGCAGCATTTTT |
| *mir-5592* | ATACTGCAGCGGCCCTTACCGTTTAAT | ATAGGATCCTATTCTTGTGGGCACGCC |
